# Relationship between structural pathology and pain behaviour in a model of osteoarthritis (OA)

**DOI:** 10.1016/j.joca.2016.06.012

**Published:** 2016-11

**Authors:** L.N. Nwosu, P.I. Mapp, V. Chapman, D.A. Walsh

**Affiliations:** † Arthritis Research UK Pain Centre, Clinical Sciences Building, Nottingham, NG5 1PB, UK; ‡ School of Medicine, University of Nottingham, UK; § School of Life Sciences, University of Nottingham, UK

**Keywords:** Pain behaviour, Monosodium iodoacetate, Synovitis, Knee OA, Structural pathology

## Abstract

**Objectives:**

To address the hypothesis that different types of established osteoarthritis (OA) pain behaviours have associations with different aspects of articular pathology, we investigated the relationship between structural knee joint pathology and pain behaviour following injection of a low vs a high dose of monosodium iodoacetate (MIA) in the rat.

**Methods:**

Rats received a single intra-articular injection of 0.1 mg or 1 mg MIA or saline (control). Pain behaviour (hind limb weight bearing asymmetry (WB) and hindpaw withdrawal threshold (PWT) to punctate stimulation) was assessed. Cartilage and synovium were examined by macroscopic visualisation of articular surfaces and histopathology.

**Results:**

Both doses of MIA lowered PWTs, 1 mg MIA also resulted in WB asymmetry. Both doses were associated with cartilage macroscopic appearance, proteoglycan loss, abnormal chondrocyte morphology, increased numbers of vessels crossing the osteochondral junction, synovitis and macrophage infiltration into the synovium. PWTs were more strongly associated with chondrocyte morphology, synovitis and macrophage infiltration than with loss of cartilage surface integrity.

**Conclusions:**

Both pain behaviours were associated with OA structural severity and synovitis. Differences in pain phenotype following low vs higher dose of MIA were identified despite similar structural pathology. OA structural pathology as traditionally measured only partially explains the MIA-induced pain phenotype.

## Introduction

Osteoarthritis (OA) is a chronic debilitating disease affecting around 8.8 million people in the UK^1^. Pain is the commonest clinical symptom that leads OA sufferers to seek medical care. OA pain contributes to loss of joint function, disability and reduced quality of life in the ageing population^2^, and is an important unmet clinical need. Our incomplete knowledge of the sources of OA pain, and the relationship between pain phenotypes and pathology hinders progress in the identification of better targets for treatments which improve OA pain.

The classification of radiographic OA depends on the presence of osteophytes and joint space narrowing (JSN). Radiographic evidence of OA is associated with pain^3^, ^4^, but this association is often only weak^5^, ^6^. Some patients report pain despite minimal radiographic changes^7^, whilst others with abnormal knee radiographs report no pain^8^. Magnetic resonance imaging (MRI) has revealed associations of bone marrow lesions or synovitis with pain^9^, ^10^, ^11^ although their contributions as direct sources of OA pain remain uncertain^5^. Central sensitization can moderate the link between joint damage and pain, through complex pain-amplifying neuroplastic alterations to the central nervous system^12^.

Preclinical models of knee OA have potential to extend understanding of OA pain mechanisms, and the contributions of specific structural features to OA pain. Intra-articular injection of the glycolysis inhibitor monosodium iodoacetate (MIA) into the rat tibiofemoral joint produces cartilage and subchondral bone pathology that are similar to those seen in human OA knees^4^, ^5^. Intra-articular MIA injection also leads to pain-related behaviours (weight bearing (WB) asymmetry and reduced hindpaw withdrawal thresholds (PWTs) to punctate stimulation)^6^, ^7^ that resemble pain on WB and more widespread reduced pain thresholds^13^ observed in human OA. Lowered PWTs implicates a contribution of central sensitisation^14^, whereas WB asymmetry likely reflects a combination of peripheral and central sensitisation.

Identifying specific aspects of joint pathology that contribute to different OA pain phenotypes might help identify pain phenotype-specific peripheral treatment targets. We hypothesised that different types of established OA pain behaviours may have associations with different aspects of articular pathology. To address this question, we have used two doses of MIA that result in two different pain profiles in the rat and then identified associations between WB asymmetry and lowered PWTs and a range of macroscopic and histopathological changes in the knee. A secondary question addressed is whether there is delayed progression of structural pathology in the lower dose MIA model.

## Materials and methods

### Animals

Studies used male Sprague–Dawley rats (Charles River, Kent, UK) (*n* = 64) weighing 250–300 g at time of intra-articular injection. Studies were conducted in accordance with UK Home Office regulations and followed the guidelines of the International Association for the Study of Pain. Rats were housed in groups of four per cage under standard conditions with a 12 h light/dark cycle, with unlimited access to food and water. Rats, anaesthetised with isoflurane (2% in O_2_), received a single intra-articular injection of MIA (0.1 mg/50 μl or 1 mg/50 μl, based on previous studies^14^, ^15^, ^16^) in sterile 0.9% normal saline through the infrapatellar ligament of one knee. In two separate experiments (experiment 1 = 24 rats, experiment 2 = 40 rats), rats were randomly assigned to the experimental (MIA) and control groups (saline) and results from both studies combined. The groups included 1 mg MIA; *n* = 18, 0.1 mg MIA; *n* = 18 and saline-injected rats; *n* = 8 stopped at day 20 and 0.1 mg MIA; *n* = 10 and saline-injected rats; *n* = 10, stopped at day 42 (Supplementary Fig. 1). Rats were killed by an overdose of carbon dioxide and tissues harvested at 20 days (1 mg; *n* = 18 and 0.1 mg MIA; *n* = 18) or 42 days (0.1 mg MIA; *n* = 10) post-injection. Previous studies indicated that OA pathology and pain behaviour were fully developed by 20 days after intra-articular injection of 1 mg MIA^17^. All outcome measurements were carried out by an experimenter blinded to intra-articular injections.

### Behavioural measurements of OA pain

Pain behaviours were measured as withdrawal thresholds (g) to punctuate stimulation of the hind paw^18^ and as hind limb weight-bearing asymmetry. Weight-bearing asymmetry was assessed as difference between hind limbs as a percentage of total weight borne through both hind limbs^19^. Measurements were obtained immediately prior to intra-articular injection (day 0) and at regular intervals from day 3–20 (1 mg or 0.1 mg MIA) or day 42 (saline and 0.1 mg MIA).

### Joint pathology

Synovia with patellae from both knees were harvested at sacrifice and immediately embedded in optimal cutting temperature (OCT) and snap frozen over melting isopentane. Tibiofemoral joints were then isolated and dissected to assess the severity of damage to the chondral surfaces. Macroscopic lesions were graded using the method of Guingamp^15^; grade 0 = normal appearance, 1 = slight yellowish discolouration of the chondral surface, 2 = little cartilage erosion in load bearing areas, 3 = large erosions extending down to the subchondral bone and 4 = large erosions with large areas of subchondral bone exposure. Five chondral compartments of the knee: femoral groove, medial and lateral femoral condyles and medial and lateral tibia plateaus were scored then summated to give a maximum possible score of 20.

Following macroscopic scoring, joints were fixed in neutral buffered formalin for 48 h, and then decalcified in 10% formic acid-formalin for 7 days at room temperature, split into anterior and posterior blocks, and embedded in paraffin. Six frontal sections per rat (three anterior and three posterior) were stained with haematoxylin and eosin (H&E) and corresponding consecutive sections for Safranin-O-Fast green^20^. Chondropathy and chondrocyte morphology were scored on H&E sections whereas proteoglycan content of the cartilage was scored on Safranin-O Fast green stained sections. Chondropathy was scored using the Janusz method as previously described^21^. It was evaluated from 1 (minimal superficial damage) to 5 (severe full thickness degeneration to tidemark). This score was multiplied by the extent of cartilage area involved (1/3, 2/3 or 3/3). Chondrocyte morphology and proteoglycan content of the cartilage were evaluated using the modified Mankin score as previously described using a Zeiss Axioscop-50 microscope (Carl Zeiss Ltd, Welwyn Garden City, UK) at 4× objective lens^22^. Chondrocyte morphology was scored from 0 (normal) to 3 (complete chondrocyte death or hypocellularity) and proteoglycan content from 0 (no loss) to 4 (complete loss of proteoglycan). Other histological assessments used a 20× objective lens. Osteochondral junction integrity was assessed as the number of vascular channels present in the articular cartilage per length of tibial plateau section (number per mm) on H&E sections^23^.

Inflammation was assessed as joint swelling (knee diameter) (mm) using digital callipers (Miyutoyo UK Ltd., Andover, UK)^24^, synovitis score and macrophage infiltration into the synovium. Synovial sections (5 μm) were either stained with H&E to assess lining thickness and cellularity from a scale of 0 (lining layer, 1–2 cells thick) to 3 (lining layer >9 cells thick and/or severe increase in cellularity)^23^. Macrophage infiltration was visualised by immunohistochemistry using the monoclonal antibody ED1 directed to CD68^25^, avidin–biotin–peroxidase conjugate (ABC) and developed with diaminobenzidine using the glucose oxidase/nickel-enhanced method^26^. Macrophage fractional area was the percentage of synovial section area immunoreactive for CD68 from four fields view on one section per rat analysed using a Zeiss Axioscop-50 microscope (Carl Zeiss Ltd, Welwyn Garden City, UK) and a KS300 image analysis system (Image Associates, Thame, UK)^26^.

### Reagents

Monoclonal antibody to CD68 (clone ED1) was from Serotec (Oxford, UK). Biotinylated rat-adsorbed horse anti-mouse antibody and avidin–biotin complexes (Vectastain^®^ Elite ABC Kits) from Vector laboratories (Peterborough, UK). Other reagents used were from Sigma–Aldrich UK.

### Statistical analysis

Data were analysed using Prism v6 (GraphPad, San Diego, California, USA). Tests for normal distribution were made using the Kolmogorov–Smirnov test and were found to be non-parametric. Therefore, groups were compared using the Kruskal–Wallis test followed by *post hoc* Dunn's tests. Pain behaviours were analysed using area under the curve (AUC) for the comparison data between arthritic and non-arthritic. Associations were evaluated between either pain behaviour endpoint measured on the day of sacrifice and joint pathology in all groups of rats and then in the MIA injected rats using univariate and multivariable linear regressions (Statistical package for the Social Sciences v.22 (SPSS Inc., Chicago, Illinois, USA)). Numerical and graphical data are presented as mean ± 95% confidence interval to denote statistical uncertainty of estimates. Association data are presented as unstandardized *β* coefficients and 95% CI. A two-tailed *P* value of less than 0.05 was taken as significant. A standard method of assessing whether the 95% CIs of the regressions contained zero was used and the null hypothesis of the means being significantly different was rejected if it did contain zero^27^.

## Results

### Dose specific effects of MIA on pain behaviour and not altered over time

**Fig. 1:**
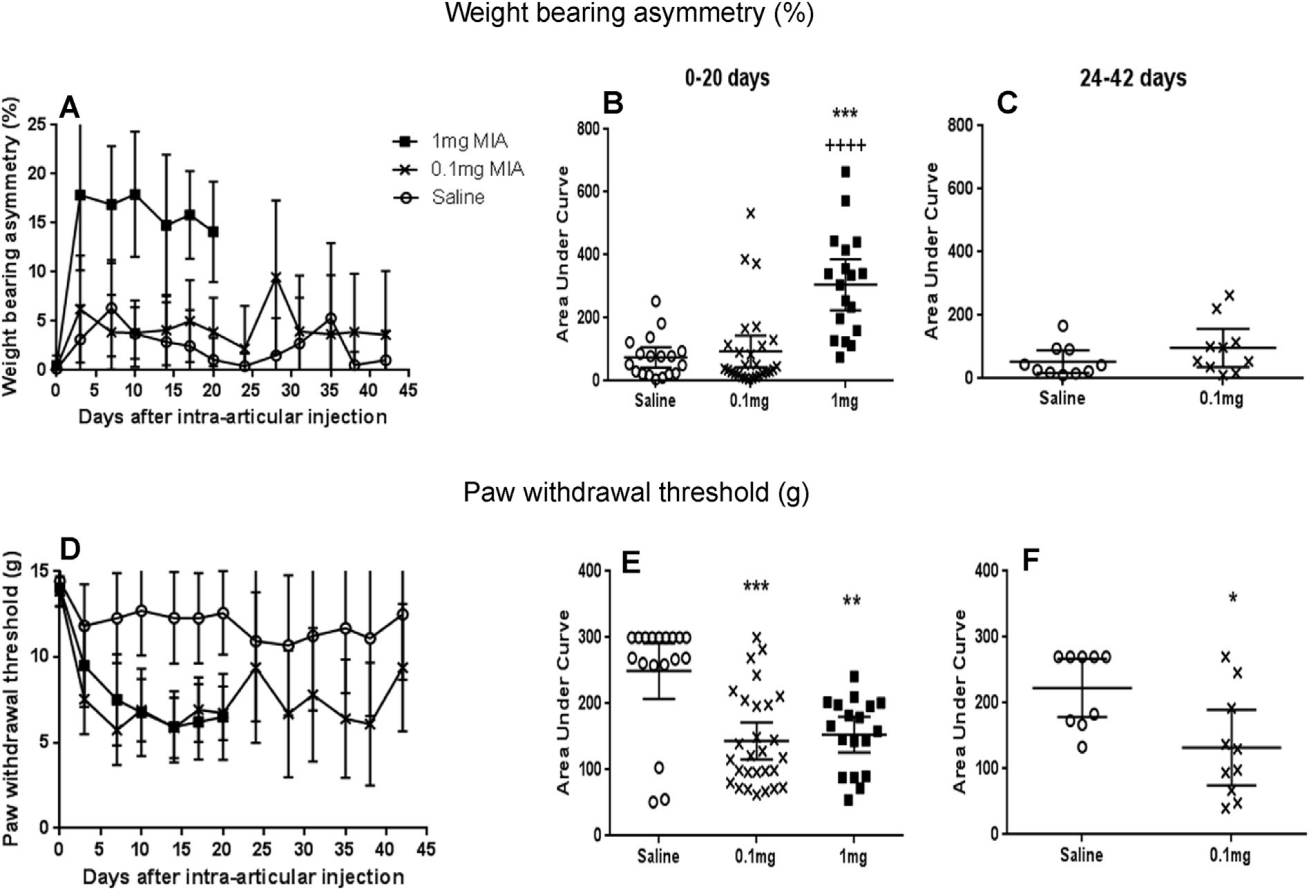
**Effect of MIA on pain behaviour**. Intra-articular injection of 1 mg MIA-induced pain behaviour measured as both increased hind-limb WB asymmetry (A and B) and reduced PWT to punctuate stimulation (D and E). Intra-articular injection of 0.1 mg MIA resulted in reduced PWTs (D–F) without significant increases in WB asymmetry (A–C). WB asymmetry was significantly greater following 1 mg MIA than following 0.1 mg MIA (B). Data indicate mean ± 95% confidence interval for *n* = 10–18 rats per group. Differences between groups were analysed using area under the curve for 0–20 days (B and E) and 24–42 days (C and F) then Kruskal–Wallis test followed by *post hoc* Dunn's tests. Significance of *post hoc* tests are denoted by the number of symbols, e.g.,: *: *P* < 0.05; **: *P* < 0.01; ***: *P* < 0.001. Asterisks (*) denote significant differences from saline-injected non-arthritic controls. Plus (+) signs denote significant differences from 0.1 mg MIA injected arthritic rats.

Intra-articular injection of the low dose of MIA did not significantly alter WB asymmetry compared with saline control, at either the early (day 0–20, *P* > 0.99) or the later time points studied (days 24–42) (0.1 mg MIA; 76.5 [31.3–141.5], saline: 34.5 [18.3–92.8] AUC, *P* = 0.17) [Fig. 1(A) and (C)]. Intra-articular injection of 1 mg of MIA was associated with WB asymmetry tested between days 0 and 20 (320 [152–422] AUC) compared with saline-injected rats (66 [22–101] AUC, *P* < 0.001) and rats injected with 0.1 mg MIA (38 [21–113] AUC, *P* < 0.001) [Fig. 1(A) and (B)]. Intra-articular injection of both doses of MIA resulted in lowered PWTs between days 0 and 20 (0.1 mg; 120 [84–203] AUC, *P* < 0.001, 1 mg; 162 [90–199] AUC, *P* = 0.004), compared with the saline-injected rats (285 [259–300]) [Fig. 1(D) and (E)]. PWTs remained lowered at the later time points (days 24–42) following injection of the lower dose (0.1 mg) of MIA (114 [62.3–205.5] AUC), compared to saline-injected rats (270 [170–270] AUC, *P* = 0.01) [Fig. 1(D) and (F)].

### MIA-induced knee inflammation

**Fig. 2:**
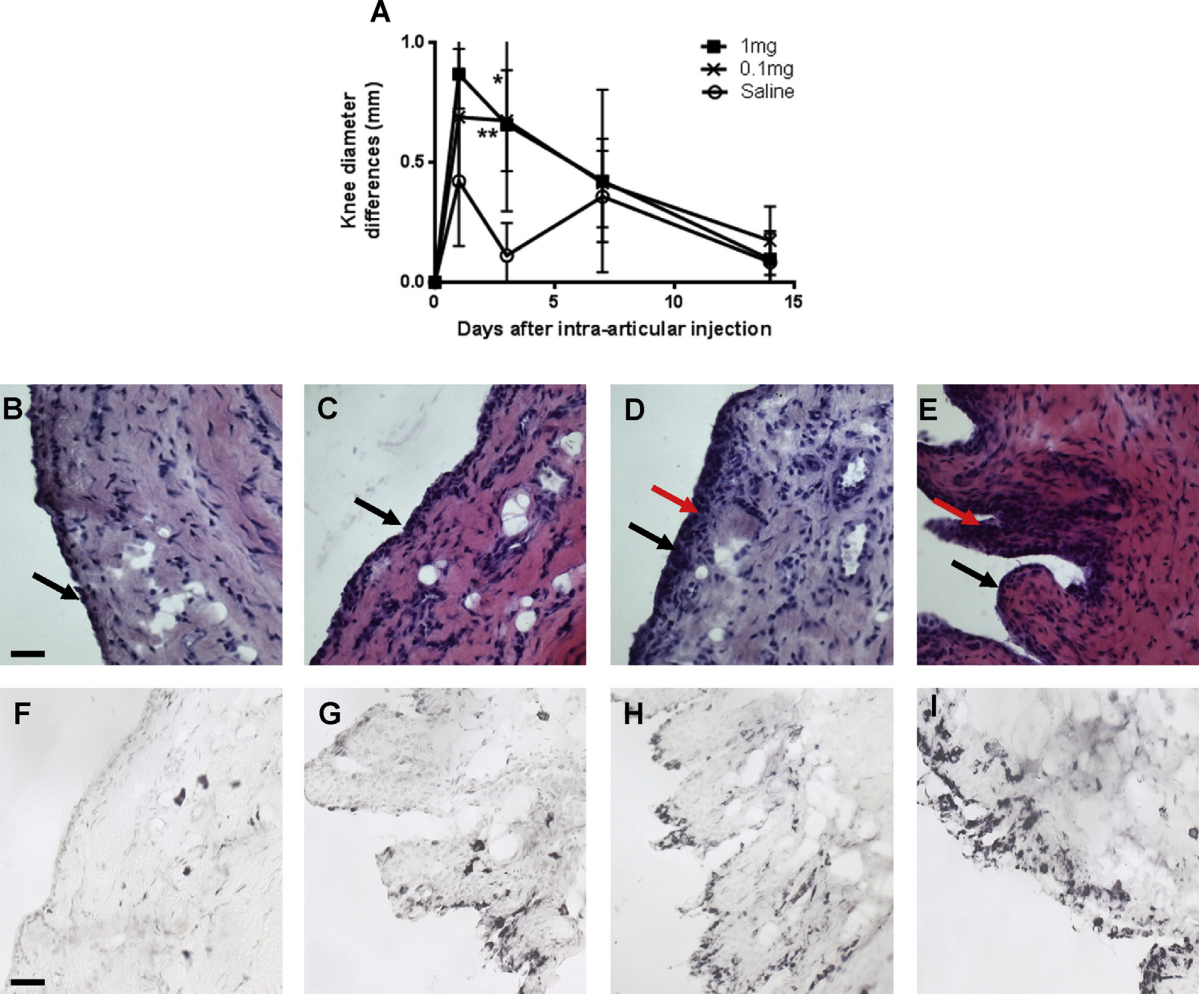
**Evidence of inflammation following OA induction**. Small changes in knee diameter were observed after intra-articular injection of MIA or saline, as indicated by differences between injected and contralateral knees (A). Knee diameter differences between injected and non-injected contralateral knees were greater in MIA injected rats compared to the saline-injected rats at day 3 after MIA injection (A). Panels B–E show synovium with H&E stain and F—I show synovium with an immunohistochemical (ED1) stain. Synovitis was characterised as synovial lining thickness/cellularity (B–E) and macrophage infiltration (F–I). Extensive synovial hyperplasia (red arrows) was apparent in the d20-1 mg and d42-0.1 mg MIA-induced OA rats (D & E) compared with non-arthritic saline controls (B). Macrophage infiltration (indicate by black staining for immunoreactivity with ED1; F—I) was higher for the d20-1 mg and d42-0.1 mg rats (H & I) compared to non-arthritic saline controls (F). Black arrows indicate synovial surface. Photomicrographs show H&E stained sections of synovial tissue from a rat with the median synovitis score from each group. Scale bars = 50 μm. Data indicate mean ± 95% confidence interval for *n* = 10–18 rats per group. Differences between groups were analysed using Kruskal–Wallis test followed by *post hoc* Dunn's tests. Significance of *post hoc* tests are denoted by the number of symbols, e.g.,: *: *P* < 0.05; **: *P* < 0.01. Asterisks (*) denote significant differences from saline-injected non-arthritic controls (A).

Both doses of MIA resulted in an acute and significant increase in ipsilateral knee diameter at 3 days post-injection, compared to the saline-injected rats [Fig. 2(A)]. Knee diameters did not differ significantly between the two doses of MIA (Fig. 2). By day 14, there were no significant differences in knee diameter between the MIA groups and the saline groups (Fig. 2).

At 20 days post-injection, inflammation grade and macrophage fractional area of the synovium were significantly increased in groups of rats injected with either 0.1 mg or 1 mg MIA, compared with saline-injected controls (Table I). There were no significant differences between the synovial inflammation grade, nor macrophage fractional area, for the two doses of MIA studied. At the later timepoint (day 42), synovial inflammation and increased macrophage fractional area remained significantly increased following intra-articular injection of 0.1 mg MIA, compare to saline-injected controls (Table I).

### Cartilage and bone changes following MIA injection

**Fig. 3:**
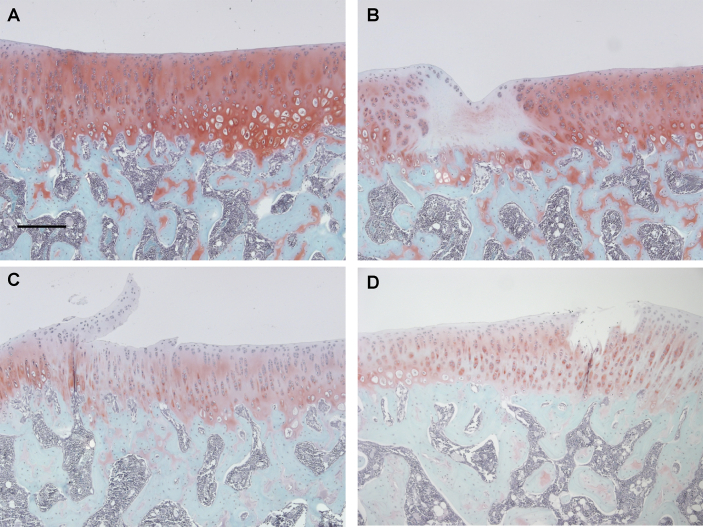
**Articular cartilage pathology 20 and 42 days after OA induction**. Histological images of the tibial plateau (A–D). Joints were sectioned in a frontal plane and stained with Safranin O-Fast green and corresponding consecutive sections stained with H&E. A; saline-treated control showing smooth cartilage and normal joint margin and chondrocyte morphology. B, C; 1 mg and 0.1 mg MIA-injected rats at day 20 and D; 0.1 mg MIA-injected rat at day 42 show degeneration of the cartilage. Proteoglycan loss (B–D) and chondrocyte cloning (B) are also present in the arthritic cartilage. Scale bars = 200 μm. Images are of knees with median cartilage surface integrity scores from each group.

Changes to the articular cartilage and subchondral bone were measured as pathological features of OA (Fig. 3 and Table I). Irrespective of the dose of MIA used, or the duration of the model, all groups which received intra-articular injection of MIA showed OA structural changes at both timepoints (Table I). Macroscopic chondropathy and histological evidence of cartilage damage and abnormal chondrocyte morphology were each higher in MIA-injected rats, compared to saline-injected controls. At 20 days post-injection, both cartilage damage and abnormal chondrocyte morphology were comparable for the two doses of MIA studied. However, proteoglycan loss only reached statistical significance for the 1 mg MIA group, compared to the saline-injected controls (Table I). There was no evidence that the period of exposure to MIA was a factor as measures of OA structural severity were comparable between 20 and 42 days following intra-articular injection of 0.1 mg MIA. The only exception to this was that the numbers of vascular channels penetrating into articular cartilage were higher at 20 days compared with 42 days post-MIA injection (Table I).

### Associations between joint pathology and pain behaviour

Using all groups of rats, WB asymmetry and PWTs were each significantly associated with macroscopic cartilage appearance and abnormal chondrocyte morphology (Table II). WB asymmetry was also significantly associated with proteoglycan loss. PWTs were significantly associated with the number of vessels crossing the osteochondral junction, and synovitis (Table II). PWTs were more strongly associated with abnormal chondrocyte morphology than with loss of cartilage surface integrity, proteoglycan loss or macrophage fractional area (Table II).

Strengths of associations with pathological features did not significantly differ between WB asymmetry or PWTs by calculating the t-statistic and comparing the two means or the 95 percent confidence intervals for the difference between the two means^27^.

When using data from only the MIA injected rats, there were no significant associations between structural OA and pain phenotypes, except for macrophage fractional area and PWTs (Table III).

Using multivariable regression models for WB asymmetry and PWTs, it was evident that only a small proportion of the variability in pain behaviours between rats was accounted by the inclusion of all of the measured histological outcome variables, as indicated by the low *R*^2^ values (WB asymmetry all *R*^2^ ≤ 0.21, PWTs all *R*^2^ ≤ 0.34). This observation suggests that other cellular or biochemical factors may contribute to the pain behaviour reported.

## Discussion

The major finding of this study is that different doses of MIA are associated with different profiles of pain behaviour and that extending the period of exposure of the low dose of MIA did not result in any further change in the magnitude or type of pain behaviour. Specifically, the lower dose of MIA was associated with lowered PWTs but no WB asymmetry at both the early or later timepoint studied (20 and 42 days), despite synovitis and osteochondral pathology comparable to rats injected with 1 mg of MIA. These data suggest that the low dose of MIA is a model of milder OA rather than more slowly progressing OA.

In our control OA group, the rapid onset and persistence of pain behaviours after injection of 1 mg of MIA into rat knees was similar to previous reports^19^, ^24^. OA structural pathology developed within 2 weeks of intra-articular injection of 1 mg MIA^28^, and resembled human OA by day 20 in the current study. The MIA model being a rapidly progressive model leads to the rapid degeneration of joint cartilage, synovitis and disruption of the underlying subchondral bone which ensues from the MIA-induced death of chondrocytes^29^. The aetiology of OA is unknown and OA in humans may not necessarily arise only due to chondrocyte death, which is evident in the MIA model^30^. A feature of the MIA model at early stages is the proliferation of cells in the outer margins which form large osteophytes at earlier time points^29^. Although we do not see the formation of osteophytes in this study owing to maybe the doses used and the time points tested, osteophytes are typical OA features and patients with OA differ in the extent of osteophytosis (e.g., previous classifications of OA as atrophic or hypertrophic). Therefore supporting the view that different animal models might reflect different human OA phenotypes^31^. Despite the disadvantages to the use of this model for studies of earlier time point mechanisms, end-stage pathology in this model is similar to human OA.

A key finding of this study is that despite similarities in the extent of joint pathology produced, the two doses of MIA are associated with different pain phenotypes, which is reminiscent of patients with OA and might reflect different balances between nociceptive and central sensitisation mechanisms to OA pain. The mechanisms that drive WB asymmetry are likely to include peripheral and central sensitization^13^. Lowered PWTs from the hindpaw are associated with spinal indices of central sensitisation in rats with OA^14^, ^32^ and may reflect a convergence of inputs from both the knee and the hind paw onto neurones in the spinal cord^33^. Additionally, at higher doses of MIA, lowered PWTs might reflect the presence of nerve damage as previously reported with the 1 mg MIA dose^34^. There is mounting evidence that central sensitisation can modulate the relationship between joint structure and OA pain both in our animal models, and in patients^35^. Our data demonstrating the presence of lowered PWTs in the absence of WB asymmetry is counter-intuitive when considering the dogma that central sensitization is at least triggered by overt nociceptive input, which is considered to be the basis for WB asymmetry. Indeed, our data indicate that, at least in rats, either pain phenotype can manifest shortly after model induction, and once developed persists throughout the experiment. A centrally augmented pain phenotype need not necessarily, as sometimes suggested^36^, be restricted to end stage OA.

We hypothesised that the relationship between pain phenotype and features of joint pathology might provide fundamental clues to the origins of OA pain. We report stronger associations of lowered PWTs with abnormal chondrocyte morphology than with proteoglycan loss or cartilage surface integrity. The primary mode of action of MIA to kill chondrocytes by inhibiting glycolysis, which likely explains the predominant associations observed between pain behaviours and chondrocyte morphology rather than other structural features observed in this study. Loss of significance in multivariable regression analysis indicates that these associations might be explained by other covariates, although possible direct mediation of pain behaviour and neuronal sensitisation by chondrocyte products deserves further study^37^. Indeed, chondrocytes can produce neuronal sensitisers such as nerve growth factor (NGF) in humans^38^ supporting possible direct contributions of chondrocytes to OA pain.

Both pain phenotypes were each similarly associated with the various structural features of OA, including macroscopic cartilage appearance, abnormal chondrocyte morphology and macrophage fractional area although not all associations reached statistical significance in the current study. Similar to findings in human OA^39^, the proportion of variation in pain behaviour that could be explained by joint structural changes was small (∼20–35%), suggesting that other cellular or biochemical changes in the joint may account for differences in the pain phenotype generated by the two MIA doses, or that are apparent in different rats or people with OA. Analysis of arthritic rats alone, revealed that macrophage fractional area was significantly associated with lowered PWTs, suggesting a contribution of peripheral inflammatory cells within the joint to the spread of pain to the hindpaw. In view of the known association between activated macrophages with human OA pain^40^, ^41^, our preclinical observation warrants further investigation. We show associations between a model of established OA and pain behaviour, and it may also be of interest to investigate whether these associations are present in this model at earlier time points. Guzman *et al.* (2003), report chondrocyte degeneration at 1 day post 1 mg MIA injection and complete chondrocyte loss with collapse of necrotic cartilage, 5 days after MIA injection^29^. The presence of these features at earlier time points may contribute to the pain behaviour observed in the 1 mg MIA model compared to the 0.1 mg MIA model. Further studies would be required in order to confirm this.

A limitation of this study is the numbers of rats used as regression analyses using only the arthritic rats show significant associations for only one of the OA structural features. Therefore although we observed relationships between structural histopathology and pain, we cannot fully conclude that the pain observed in these rats are primarily mediated by the pathology. Increasing the numbers of arthritic rats may help with confirming the involvement of the features of OA pathology in mediating pain in this model. The relationship observed between histopathological OA features and pain explains only a small proportion of variability between rats.

In conclusion, the absence of WB asymmetry even 42 days after injection of 0.1 mg MIA indicates that this lower dose produced a discrete OA pain phenotype, rather than producing a more slowly developing OA model than that induced by 1 mg MIA injection^42^. Our study demonstrates that varying experimental procedures such as dose of MIA generates discrete OA pain phenotypes that may model pain phenotypes in human OA, which may aid the elucidation of the diverse mechanisms underlying OA pain and develop targeted treatments suitable for phenotypic subgroups.

## Author contributions

All authors were involved in drafting the article or revising it critically for important intellectual content, and all authors approved the final version to be published.

Study conception and design; Nwosu, Mapp, Chapman, Walsh.

Acquisition of data; Nwosu.

Analysis and interpretation of data; Nwosu, Chapman, Walsh.

## Competing interests

The authors have no competing interests.

## Funding

This work was supported by Arthritis Research UK, grant number 20777 and LNN studentship supported by the University of Nottingham.

## Figures and Tables

**Table I:** Pathological features in articular cartilage and subchondral bone 20 and 42 days after intra-articular injection of MIA

Structural changes (range)	D42-Saline	D20-1 mg MIA	D20-0.1 mg MIA	D42-0.1 mg MIA
Macroscopic cartilage appearance (0–20)	3.4 (1.8–5.0)	11 (8.4–13); <0.001*	9.2 (7.4–11); 0.005*	9.6 (7.7–12); 0.002*
Cartilage surface integrity (0–24)	0.67 (0.33–1)	3.2 (2.0–4.4); <0.001*	2.5 (1.9–3.2); <0.001*	3.1 (2.1–4.2); <0.001*
Abnormal chondrocyte morphology (0–3)	0.62 (0.37–0.87)	2 (1.8–2.3); <0.001*	1.9 (1.6–2.2); <0.001*	1.5 (1.0–2.0); 0.049*
Osteochondral junction integrity (vessels/mm)	0.5 (0.39–0.60)	1.1 (0.91–1.3); 0.02*, 0.02+	1.3 (1.1–1.5); <0.001*, <0.001+	0.49 (0.36–0.62)
Proteoglycan loss (0–4)	2 (1.7–2.2)	2.7 (2.5–3.0); <0.001*	2.4 (2.0–2.8)	2.2 (1.7–2.7)
Synovitis (0–3)	0.82 (0.41–1.2)	2.4 (2.1–2.7); <0.001*	1.8 (1.4–2.2)	2.7 (1.9–3.4); <0.001*
Macrophage fractional area (%)	5.4 (3.2–7.6)	14 (11–18); <0.001*	8.3 (5.8–11)	16 (11–22); <0.001*

Data are presented as mean 95% CI. Significance of *post hoc* tests is denoted by the *P* value with a symbol of either an asterisks (*) to denote significant differences from saline-injected non-arthritic controls or Plus signs (+) denote significant differences from rats 42 days after 0.1 mg MIA injection. Day 42-saline and 0.1 mg MIA (*n* = 10), day 20–1 mg and 0.1 mg MIA (*n* = 18).

**Table II:** Associations of pain behaviour phenotypes with OA structural features

	Weight bearing asymmetry (%)	Paw withdrawal threshold (g)
	*β* (95% CI); *P*	*β* (95% CI); *P*
Macroscopic cartilage appearance	**1.10 (0.24 to 1.96); 0.01**	**−0.52 (−0.98 to −0.07); 0.03**
Cartilage surface integrity	1.18 (−0.02 to 2.37); 0.053	−0.61 (−1.31 to 0.10); 0.09
Abnormal chondrocyte morphology	**3.03 (0.25 to 5.81); 0.03**	**−3.28 (−4.75 to −1.80); <0.001**
Osteochondral junction integrity	4.80 (−0.27 to 9.86); 0.06	**−3.04 (−5.60 to −0.49); 0.02**
Proteoglycan loss	**3.76 (0.47 to 7.06); 0.03**	−1.86 (−3.82 to 0.10); 0.06
Synovitis	1.29 (−0.95 to 3.52); 0.25	**−1.64 (−2.89 to −0.40); 0.01**
Macrophage fractional area	0.10 (−0.24 to 0.44); 0.54	−0.02 (−0.22 to 0.18); 0.84

Univariate associations expressed as unstandardised *β* coefficients from regression analyses of pain behaviour on the **day of sacrifice** and joint pathology in all groups of rats; *n* = 64/pathology score except macroscopic pathology and osteochondral junction integrity (*n* = 40). Data are presented as mean (95% CI) with corresponding *P* value and highlighted in bold if statistically significant.

**Table III:** Associations of pain behaviour phenotypes with OA structural features in MIA injected rats

	Weight bearing asymmetry (%)	Paw withdrawal threshold (g)
	*β* (95% CI); *P*	*β* (95% CI); *P*
Macroscopic cartilage appearance (n = 52)	1.08 (−0.54 to 2.71); 0.18	−0.16 (−0.87 to 0.56); 0.66
Cartilage surface integrity (n = 102)	0.53 (−1.12 to 2.18); 0.52	0.52 (−0.27 to 1.30); 0.19
Abnormal chondrocyte morphology (n = 76)	0.65 (−3.12 to 10.02); 0.29	−1.32 (−3.73 to 1.08); 0.27
Osteochondral junction integrity (n = 60)	3.45 (−0.41 to 3.46); 0.12	−1.36 (−4.19 to 1.47); 0.33
Proteoglycan loss (n = 76)	2.79 (−1.59 to 7.16); 0.21	−0.31 (−2.45 to 1.83); 0.77
Synovitis (n = 102)	−0.31 (−3.79 to 3.16); 0.86	0.41 (−1.26 to 2.07); 0.63
Macrophage fractional area (n = 55)	−0.10 (−0.54 to 0.34); 0.65	**0.24 (0.03 to 0.44); 0.03**

Univariate associations expressed as unstandardised *β* coefficients from regression analyses of pain behaviour on the **day of sacrifice** and joint pathology in arthritic rats only; *n* = 46/pathology score except macroscopic pathology and osteochondral junction integrity (*n* = 30). Data are presented as mean (95% CI) with corresponding *P* value and highlighted in bold if statistically significant.

## Appendix A Supplementary data

Supplementary Fig. 1 **Time-course and study design of experiments**. Rats weighing 250–300 g were habituated to pain behaviour rooms and testing equipments (incapacitance meter for weight bearing assessments and von Frey boxes for von Frey measurements) 2 days prior to the start of the study. Baseline weights, knee diameter (mm) and pain behaviour data were obtained on day 0. Following this, rats were anaesthetised and given a single 50 μl intra-articular injection of saline, 0.1 mg or 1 mg MIA. In two separate experiments rats were randomly placed into groups of saline, 0.1 mg MIA and 1 mg MIA. **Experiment 1** consisted of eight rats per group (*n* = 24), and the experiment stopped at 20 days post intra-articular injection. **Experiment 2** consisted of 10 rats per group (*n* = 40), and the experiment stopped at 20 days post intra-articular injection for the 1 mg (10) and 0.1 mg (10) MIA dose, and at 42 days post intra-articular injection for the 0.1 mg (10) MIA and saline (10) dose. The weights of the rats and their knee diameters were measured weekly, while the pain measurements were carried out twice weekly until the end of the experiments. At the end of each timepoint, rats were killed by an overdose of CO_2_ and their tissues (synovium and knee) harvested for histology. Macroscopic scoring of knee pathology was carried out during tissue harvesting.
