# Supplementary material for: Relationship between structural pathology and pain behaviour in a model of osteoarthritis (OA)
Source: Osteoarthritis Cartilage. 2016 Nov;24(11):1910–7. doi: 10.1016/j.joca.2016.06.012 (PMC5081684; doi:10.1016/j.joca.2016.06.012)
Supplement: Supplementary Fig. 1 — Time-course and study design of experiments. Rats weighing 250–300 g were habituated to pain behaviour rooms and testing equipments (incapacitance meter for weight bearing assessments and von Frey boxes for von Frey measurements) 2 days prior to the start of the study. Baseline weights, knee diameter (mm) and pain behaviour data were obtained on day 0. Following this, rats were anaesthetised and given a single 50 μl intra-articular injection of saline, 0.1 mg or 1 mg MIA. In two separate experiments rats were randomly placed into groups of saline, 0.1 mg MIA and 1 mg MIA. Experiment 1 consisted of eight rats per group (n = 24), and the experiment stopped at 20 days post intra-articular injection. Experiment 2 consisted of 10 rats per group (n = 40), and the experiment stopped at 20 days post intra-articular injection for the 1 mg (10) and 0.1 mg (10) MIA dose, and at 42 days post intra-articular injection for the 0.1 mg (10) MIA and saline (10) dose. The weights of the rats and their knee diameters were measured weekly, while the pain measurements were carried out twice weekly until the end of the experiments. At the end of each timepoint, rats were killed by an overdose of CO2 and their tissues (synovium and knee) harvested for histology. Macroscopic scoring of knee pathology was carried out during tissue harvesting. [file mmc1.pptx]

## Slide 1
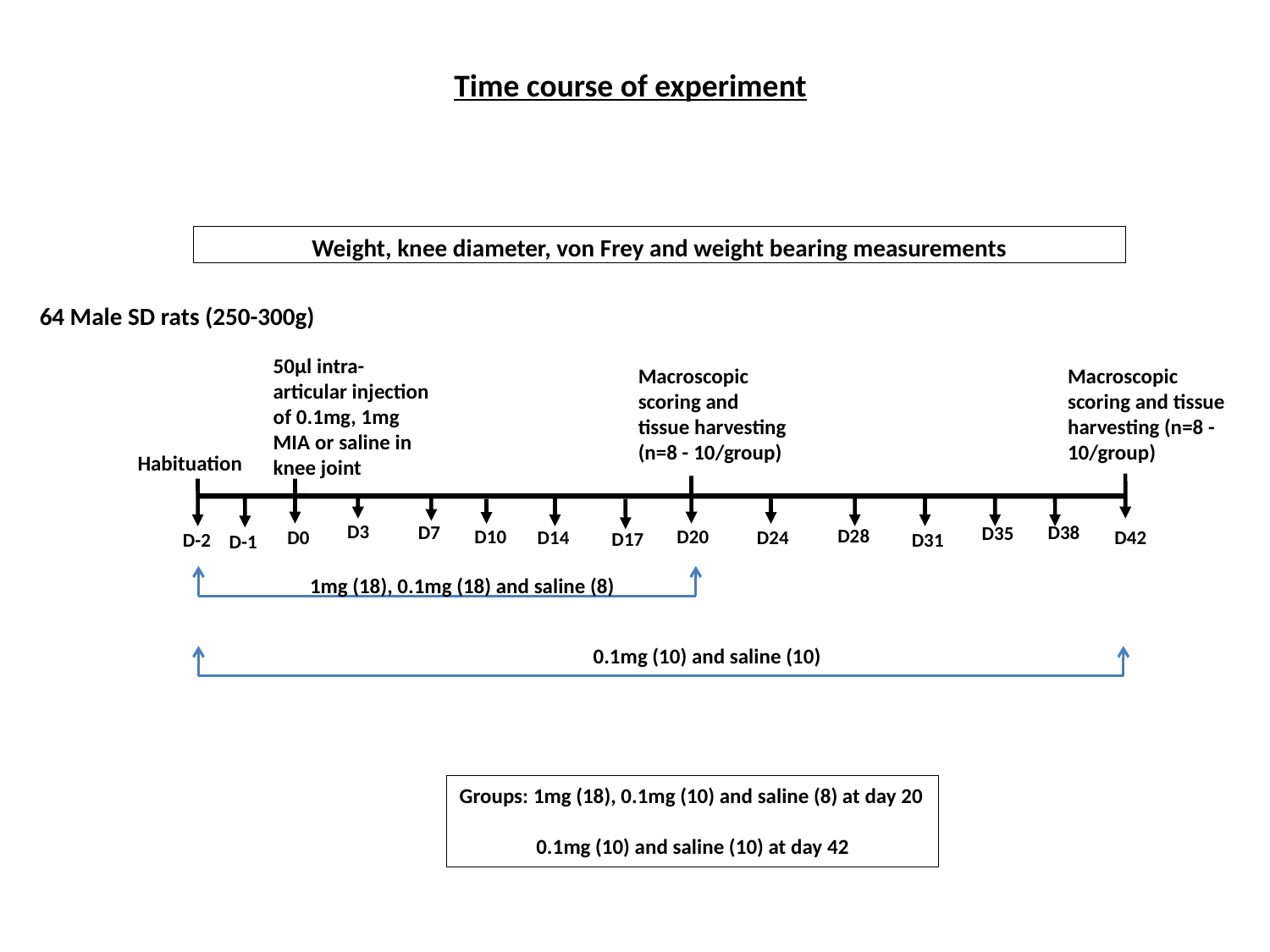

Time course of experiment
Weight, knee diameter, von Frey and weight bearing measurements
64 Male SD rats (250-300g)
50µl intra-articular injection of 0.1mg, 1mg MIA or saline in knee joint
Macroscopic scoring and tissue harvesting (n=8 - 10/group)
Macroscopic scoring and tissue harvesting (n=8 -10/group)
Habituation
D3
D38
D7
D35
D28
D20
D10
D42
D14
D0
D24
D17
D-2
D31
D-1
1mg (18), 0.1mg (18) and saline (8)
0.1mg (10) and saline (10)
Groups: 1mg (18), 0.1mg (10) and saline (8) at day 20
0.1mg (10) and saline (10) at day 42
